# AMP-activated Protein Kinase Controls Immediate Early Genes Expression Following Synaptic Activation Through the PKA/CREB Pathway

**DOI:** 10.3390/ijms19123716

**Published:** 2018-11-22

**Authors:** Sébastien Didier, Florent Sauvé, Manon Domise, Luc Buée, Claudia Marinangeli, Valérie Vingtdeux

**Affiliations:** Université de Lille, Inserm, Centre Hospitalo-Universitaire de Lille, UMR-S1172—JPArc—Centre de Recherche Jean-Pierre AUBERT, F-59000 Lille, France; sebastien.didier1@gmail.com (S.D.); florent.sauve@inserm.fr (F.S.); manon.domise@inserm.fr (M.D.); luc.buee@inserm.fr (L.B.); c.marinangeli83@gmail.com (C.M.)

**Keywords:** AMPK, synaptic activation, PKA, CREB, soluble Adenylyl cyclase, Immediate early genes, transcription

## Abstract

Long-term memory formation depends on the expression of immediate early genes (IEGs). Their expression, which is induced by synaptic activation, is mainly regulated by the 3′,5′-cyclic AMP (cAMP)-dependent protein kinase/cAMP response element binding protein (cAMP-dependent protein kinase (PKA)/ cAMP response element binding (CREB)) signaling pathway. Synaptic activation being highly energy demanding, neurons must maintain their energetic homeostasis in order to successfully induce long-term memory formation. In this context, we previously demonstrated that the expression of IEGs required the activation of AMP-activated protein kinase (AMPK) to sustain the energetic requirements linked to synaptic transmission. Here, we sought to determine the molecular mechanisms by which AMPK regulates the expression of IEGs. To this end, we assessed the involvement of AMPK in the regulation of pathways involved in the expression of IEGs upon synaptic activation in differentiated primary neurons. Our data demonstrated that AMPK regulated IEGs transcription via the PKA/CREB pathway, which relied on the activity of the soluble adenylyl cyclase. Our data highlight the interplay between AMPK and PKA/CREB signaling pathways that allows synaptic activation to be transduced into the expression of IEGs, thus exemplifying how learning and memory mechanisms are under metabolic control.

## 1. Introduction

Long-term memory formation as well as long lasting forms of synaptic plasticity depend on the expression of new genes and proteins. These activity-regulated genes, referred to as immediate early genes (IEGs), encode for transcription factors and proteins that have the potential to transduce synaptic activity directly into immediate changes of neural function. They include, for example, *Arc/Arg3.1*, *EgrI/Zif268*, and *c-Fos*. These genes are indirect markers of neuronal activity and are used to map neuronal networks and circuits engaged in information processing and plasticity [1]. For instance, Arc (activity-regulated cytoskeleton-associated protein) is a cytosolic protein found in post-synaptic densities that regulates the endocytosis of AMPA receptors [2], Notch signaling, spine density, and morphology [3] through actin remodeling [4]. *Arc* knock-out (KO) mice display impairments in the formation of long-term memories while short-term memory is not affected [5]. EgrI/Zif268 and c-Fos interact with an array of other transcription factors to regulate gene expression. *EgrI* and *c-Fos* KO animals display deficits in complex behavioral tasks and memories [6,7].

Signaling pathways involved in activity-driven regulation of transcription and translation have been the object of many studies, however, not all the components have been elucidated. One of the most studied mediators of these transcriptional changes is the transcription factor 3′,5′-cyclic AMP (cAMP) response element-binding (CREB) protein [8]. Indeed, many of the IEGs contain cAMP response elements (CRE) and thus are regulated by the transcription factor CREB. CREB signaling is regulated by phosphorylation on its Ser^133^, a key regulatory site where phosphorylation ensures the transcriptional function of CREB [9,10]. While several signaling pathways and kinases are known to induce CREB phosphorylation, the most important CREB kinase is the 3′,5′-cyclic AMP (cAMP)-dependent protein kinase (PKA). PKA activity, in turn, is known to be regulated upstream by signaling pathways leading to the increase of intracellular cAMP levels, and thus by the activity of adenylyl cyclases (ACs), the best characterized of which being the G protein-coupled receptors (GPCRs) [11].

Altogether, these processes are induced by synaptic activation and in particular by glutamatergic neurotransmission. Importantly, glutamatergic transmission is a highly energy-consuming process [12,13]. Within neurons, energy levels are regulated by the AMP-activated protein kinase (AMPK). AMPK is a Ser/Thr protein kinase, which is an important intracellular energy sensor and regulator. AMPK is composed of a catalytic subunit α and two regulatory subunits β and γ [14]. AMPK activity is regulated by the intracellular levels of adenine nucleotides AMP and ATP [15,16] and by the phosphorylation of its α subunit on Thr^172^ [17,18,19]. Interestingly, we recently reported that AMPK was necessary to maintain energy levels in neurons during synaptic activation [20]. Indeed, following glutamatergic synaptic stimulation we showed that AMPK activity was necessary to up-regulate glycolysis and mitochondrial respiration in order to maintain ATP levels within neurons. Failure to maintain energy homeostasis, through AMPK inhibition, prevented IEGs protein expression, synaptic plasticity, and hence long-term memory formation. This evidence strongly suggested that AMPK might act as a gatekeeper inside the neurons to allow signal transduction only in conditions where energy supplies are sufficient.

The goal of the present study was to determine the signaling pathway regulated by AMPK that allows the expression of IEGs. To this end, synaptic activation was induced in primary neurons, and AMPK and PKA signaling pathways were studied in these conditions. Our results showed that both signaling pathways were required for the expression of IEGs to occur. Interestingly, we also showed that the soluble adenylyl cyclase (AC) was responsible for PKA activation. Finally, inhibition of AMPK led to a downregulation of PKA pathway activation. Altogether, these data show how AMPK and PKA pathways interplay to regulate the expression of IEGs following synaptic activation.

## 2. Results

### 2.1. AMPK Activity is Required for Synaptic Activity-Induced IEGs Transcriptional Regulation

**Figure 1:**
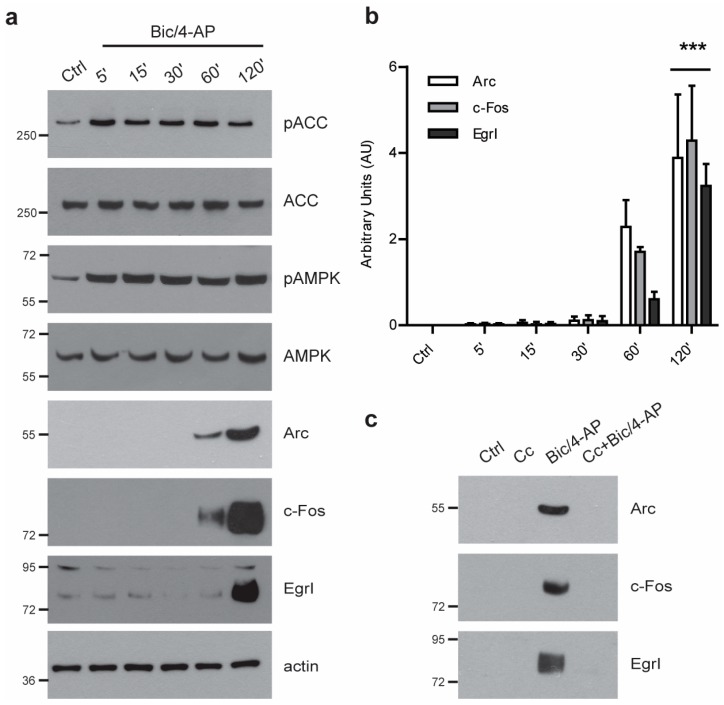
AMP-activated protein kinase (AMPK) is required for the expression of immediate early genes (IEGs) following glutamatergic activation. (**a**) Primary neurons at 15 days in vitro (DIV) treated with bicuculline and 4-aminopyridine (Bic/4-AP) (50µM/2.5mM) for the indicated time were subjected to immunoblotting with anti- phospho-AMPK (pAMPK), phospho-acetyl-CoA carboxylase (pACC), acetyl-CoA carboxylase (ACC), total AMPK, Arc, c-Fos, EgrI, and actin antibodies. Results are representative of at least four experiments. (**b**) Quantification of Western blot (WB) as in (**a**) showing Arc, c-Fos, and EgrI expression. Results show mean ± SD (*n* = 4). One-way ANOVA followed by Bonferroni’s post-hoc test were used for evaluation of statistical significance, * *p* < 0.05, *** *p* < 0.001 compared to control condition. (**c**) Primary neurons at 15 DIV were pre-treated for 20 min in presence or absence of the AMPK inhibitor Compound C (Cc, 10 µM) prior to being treated with Bic/4-AP (50µM/2.5mM, 2 h). Cell lysates were subjected to immunoblotting with anti-Arc, c-Fos, EgrI, and actin antibodies. Results are representative of at least four experiments.

In order to determine the signaling pathway regulated by AMPK that allows for the expression of IEGs, we used primary neuronal cultures at 15 days in vitro (DIV) in which glutamatergic synaptic activation was induced using bicuculline and 4-aminopyridine (Bic/4-AP) as previously described [20,21,22]. As we recently showed, synaptic activation (SA) in this model led to the rapid activation of AMPK, as indicated by the increased phosphorylation of AMPK at Thr^172^, and of its direct target, the Acetyl-CoA carboxylase (ACC), at Ser^79^. Additionally, after 2 h of SA, a significant increase of the IEGs Arc, EgrI, and c-Fos expression was observed (Figure 1a, b). Further, AMPK inhibition using Compound C (Cc) prevented the expression of IEGs following SA (Figure 1c). These data demonstrated, as we previously reported [20], that proper AMPK activation is necessary for the expression of IEGs.

**Figure 2:**
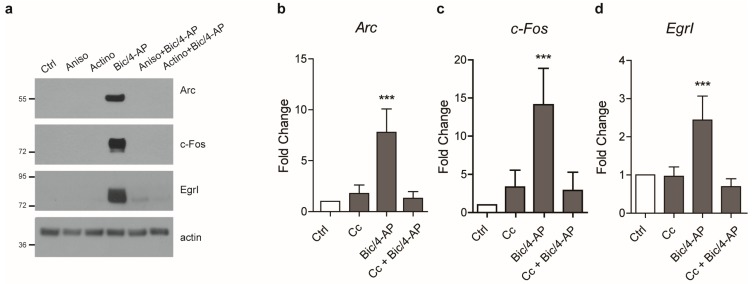
Expression of IEGs required *de novo* messenger RNA (mRNA) transcription and translation. (**a**) Primary neurons at 15 DIV co-treated with Bic/4-AP (50µM/2.5mM) and the translation inhibitor anisomycin A (Aniso, 25 µM) or the transcription inhibitor actinomycin D (Actino, 10 µM) for 2 h were subjected to immunoblotting with anti-Arc, c-Fos, EgrI, and actin antibodies. Results are representative of at least four experiments. Results demonstrate that both translation and transcription are required for the expression of IEGs following synaptic activation (SA). (**b**–**d**) mRNA levels of *Arc*, *c-Fos*, and *EgrI* were determined by quantitative PCR in primary neurons at 15 DIV treated with Bic/4-AP (50µM/2.5mM, 30 min) after 20 min with or without pre-treatment with the AMPK inhibitor Compound C (Cc, 10 µM). Results show mean ± SD (*n* = 4–6). One-way ANOVA followed by Bonferroni’s post-hoc test were used for evaluation of statistical significance, *** *p* < 0.001.

IEGs protein expression relies on the transcription of new genes, however, it was also proposed that it could result from the translation of a pre-existing pool of messenger RNA (mRNA) that is dendritically localized [23]. Therefore, we next thought to determine whether the expression of IEGs in our system was dependent on new mRNA expression or whether a pre-existing pool of mRNA could be sufficient to allow for the expression of IEGs following SA. To this end, translation was inhibited using anisomycin A and transcription inhibited using the RNA polymerase inhibitor actinomycin D. Both anisomycin A and actinomycin D repressed the expression of IEGs’ proteins induced by SA (Figure 2a), showing that both *de novo* translation and transcription were necessary for the expression of IEGs. Altogether, these data implied that the expression of IEGs required new mRNA synthesis following SA. Indeed, Bic/4-AP stimulation led to a significant up-regulation of *Arc*, *c-Fos*, and *EgrI* mRNA (Figure 2b–d). We next assessed whether AMPK was required for this increased transcription to occur. Pre-treatment with the AMPK inhibitor Cc prevented the expression of IEGs, demonstrating that AMPK repression led to an inhibition of the activity-mediated IEG’s mRNA levels of induction (Figure 2b–d). Altogether, these results showed that AMPK activity is involved in the transcriptional regulation of IEGs.

### 2.2. PKA Pathway Is Activated Following SA and Is Required for The Expression of IEGs

**Figure 3:**
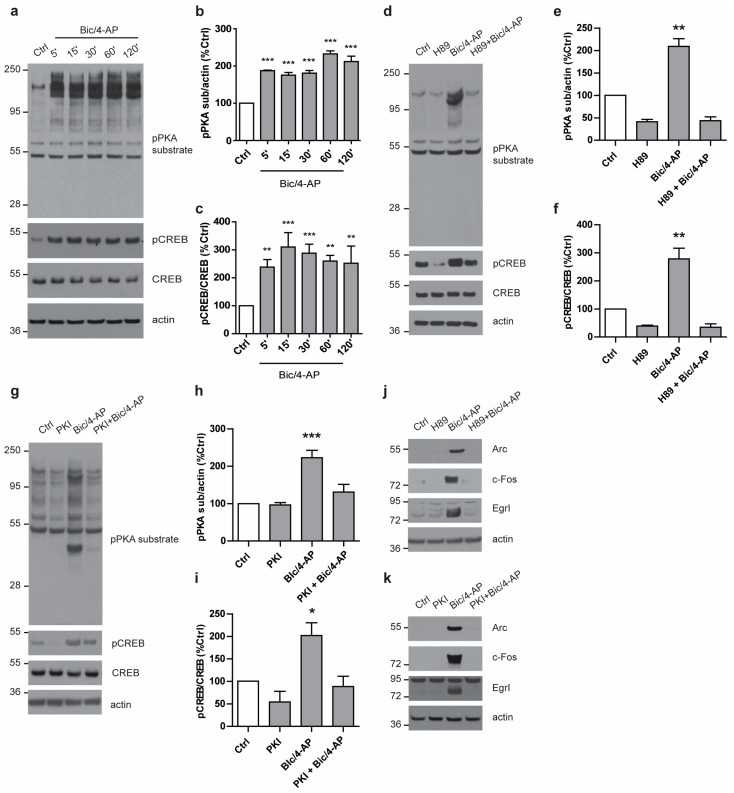
The cAMP-dependent protein kinase (PKA) pathway is rapidly activated following synaptic activation and is required for the expression of IEGs. (3′,5′-cyclic AMP = cAMP) (**a**) Primary neurons at 15 DIV treated with Bic/4-AP (50µM/2.5mM) for the indicated times were subjected to immunoblotting with anti- phospho-PKA substrate (pPKA sub), phospho-CREB (pCREB), total CREB, and actin antibodies. (cAMP response element binding = CREB) (**b**, **c**) Quantification of WB as in (a) showing the ratios pPKA sub/actin (**b**) and pCREB/CREB (**c**) expressed as a percentage of control (*n* = 3). (**d**–**g**) Primary neurons at 15 DIV treated with Bic/4-AP (50µM/2.5mM, 10 min) with or without 20 min pre-treatment with the PKA inhibitors H89 (20 µM, **d**–**f**) or PKA peptide inhibitor (PKI) (50 µM, **g**–**i**) were subjected to immunoblotting with anti- pPKA sub, pCREB, total CREB, and actin antibodies (**d**,**i**). Quantification of WB as in (d) and (g) showing the ratios pPKA sub/actin (**e**,**h**) and pCREB/CREB (**f**,**i**) expressed as a percentage of control (*n* = 4). (**j**,**k**) Primary neurons at 15 DIV treated with Bic/4-AP (50µM/2.5mM, 2 h) with or without 20 min pre-treatment with the PKA inhibitors H89 (20 µM, **j**) or PKI (50 µM, **k**) were subjected to immunoblotting with anti-Arc, cFos, EgrI, and actin antibodies. Results are representative of at least four experiments. Results show mean ± SD. One-way ANOVA followed by Bonferroni’s post-hoc test were used for evaluation of statistical significance. * *p* < 0.05, ** *p* < 0.01, *** *p* < 0.001.

As the main pathway involved in the regulation of IEGs transcription is the PKA/CREB pathway, we questioned whether AMPK could cross-talk with this signaling pathway. We first assessed the activation of the PKA/CREB pathway following SA. To this end, we used an anti-phospho-PKA substrate antibody that detects proteins containing a phosphorylated Ser/Thr residue within the consensus sequence for PKA, thus giving an indirect readout of PKA activation status. Bic/4-AP stimulation led to a rapid and sustained activation of PKA, as observed using the anti-phospho-PKA substrate antibody as well as to the phosphorylation of CREB at Ser^133^, a direct target of PKA (Figure 3a–c). Altogether, these data demonstrated that the PKA pathway was rapidly activated following SA and led to the activation of CREB.

We next determined whether the PKA pathway was required for the expression of IEGs following SA. To this end, primary neurons were pre-treated with the pharmacological PKA inhibitor H89, prior to being treated with Bic/4-AP (Figure 3d–f). Additionally, as H89 was reported to display off-target effects, to further validate the implication of PKA, we also used PKI 14–22 amide, a specific PKA peptide inhibitor (PKI) (Figure 3g–i). Results showed that both H89 and PKI prevented the PKA-substrate and CREB phosphorylation induced by SA (Figure 3d–i). Furthermore, our results showed that PKA inhibition by H89 or PKI led to a significant reduction of the expression of IEGs (Figure 3j,k). Consistent with previous reports, the present results show that PKA activation during SA is required for the expression of IEGs.

### 2.3. PKA Activation Following Synaptic Activation is Mediated by the Soluble AC

**Figure 4:**
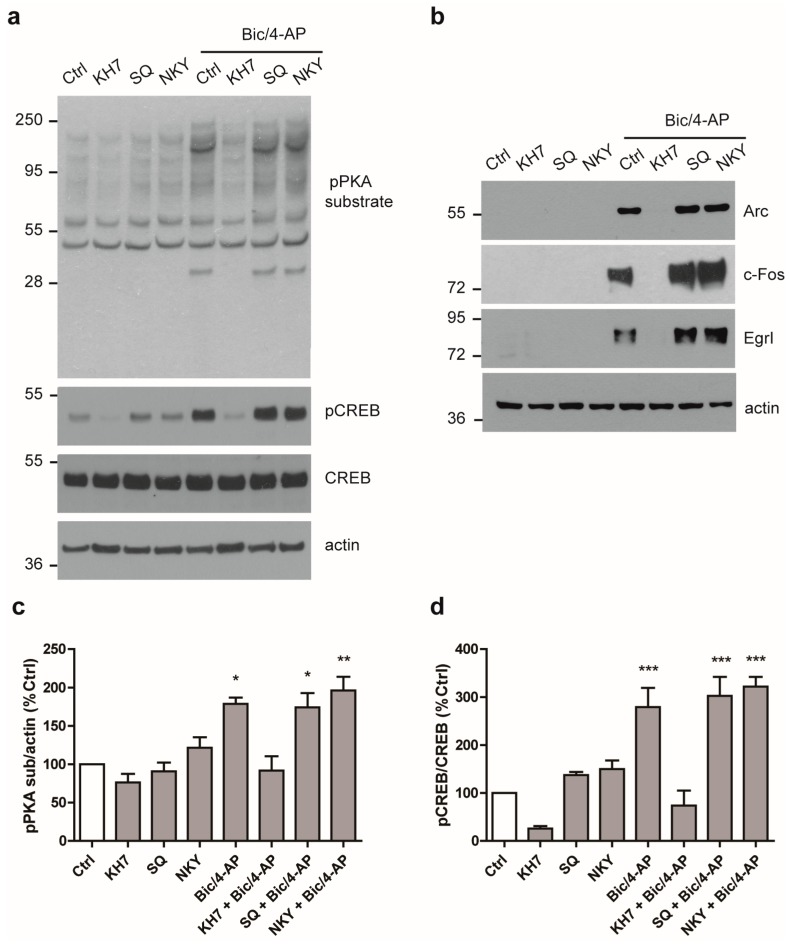
PKA activation following SA is dependent on soluble adenylyl cyclase (sAC). (**a**) Primary neurons at 15 DIV treated with Bic/4-AP (50µM/2.5mM, 10 min) with or without 20 min pre-treatment with the adenylyl cyclase (AC) inhibitors KH7 (20 µM), SQ22536 (SQ, 20 µM), and NKY80 (NKY, 20 µM) were subjected to immunoblotting with anti- phospho-PKA substrate (pPKA sub), phospho-CREB (pCREB), total CREB, and actin antibodies. (Ctrl was without pre-treatment) (**b**) Quantification of WB as in (a) showing the ratios pPKA sub/actin (c) and pCREB/CREB (d) expressed as a percentage of control (*n* = 4). (**c**) Primary neurons at 15 DIV treated with Bic/4-AP (2 h) with or without 20 min pre-treatment with the AC inhibitors KH7 (20 µM), SQ (20 µM), and NKY (20 µM) were subjected to immunoblotting with anti- Arc, c-Fos, EgrI, and actin antibodies. Results are representative of at least four experiments. Results show mean ± SD. One-way ANOVA followed by Bonferroni’s post-hoc test were used for evaluation of statistical significance. * *p* < 0.05, ** *p* < 0.01, *** *p* < 0.001.

PKA is activated by the second messenger cAMP that is produced from ATP by AC. We next determined which of the ACs were responsible for PKA activation. To this end, neurons were pre-treated with various inhibitors of ACs, including inhibitors directed against the membrane bound ACs, (SQ22536 or NKY80), or the specific inhibitor of the soluble AC (sAC) KH7 before Bic/4-AP stimulation [24]. Results showed that only KH7 inhibited PKA-substrate and CREB phosphorylation following SA (Figure 4a,c,d) and hence inhibited the expression of IEGs (Figure 4b). Thus, PKA was activated following SA-regulated expression of IEGs via the sAC activity, since only KH7 pre-treatment repressed PKA activation and the expression of IEGs.

### 2.4. AMPK Regulates PKA Activation Following SA

**Figure 5:**
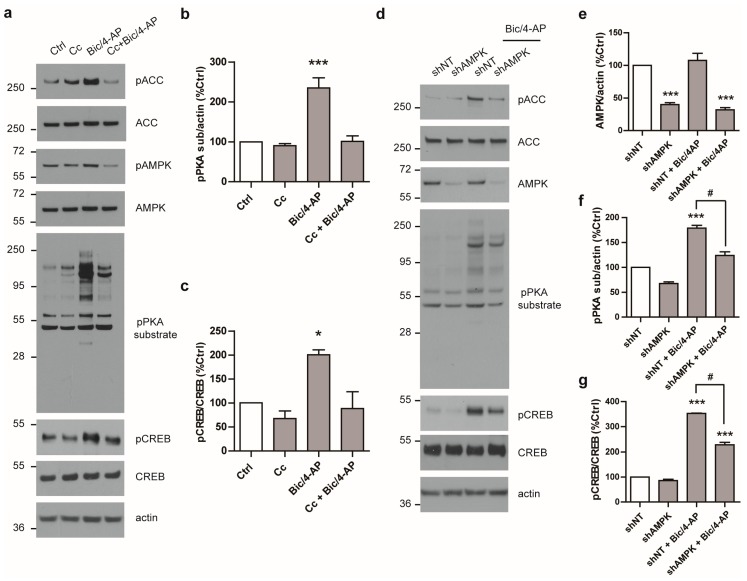
AMPK regulates PKA activation following SA. (**a**) Primary neurons at 15 DIV treated with Bic/4-AP (50µM/2.5mM, 10 min) with or without 20 min pre-treatment with the AMPK inhibitor Compound C (Cc, 10 µM) were subjected to immunoblotting with anti- phospho-AMPK (pAMPK), phospho-ACC (pACC), phospho-PKA substrate (pPKA sub), phospho-CREB (pCREB), total AMPK, ACC, CREB, and actin antibodies. (**b**,**c**) Quantification of WB as in (a) showing the ratios pPKA sub/actin (**b**) and pCREB/CREB (**c**) expressed as a percentage of control (*n* = 6). (**d**) 15 DIV primary neurons transduced for seven days with control non-targeting short hairpin RNA (shRNA) non-targeting shRNA (shNT) or with AMPK shRNA (shAMPK) were stimulated with Bic/4-AP (10 min) and subjected to immunoblotting with anti- pACC, pPKA sub, pCREB,total AMPK, ACC, CREB, and actin antibodies. (**e**,**f**,**g**) Quantification of WB as in (d) showing the ratios AMPK/actin (**e**), pPKA sub/actin (**f**), and pCREB/CREB (**g**) expressed as percentage of control (*n* = 3). Results show mean ± SD. One-way ANOVA followed by Bonferroni’s post-hoc test were used for evaluation of statistical significance. * *p* < 0.05, ** *p* < 0.01, *** *p* < 0.001 as compared to Ctrl (**b**,**c**) or shNT (**e**,**f**,**g**), # *p* < 0.05 as compared to shNT + Bic/4-AP condition (**e**,**f**).

To determine whether AMPK could be involved in the regulation of the PKA pathway, neurons were pre-treated with Cc as described above. Interestingly, Cc-pre-treatment prohibited PKA activation mediated by Bic/4-AP, as both PKA substrate and CREB were no longer phosphorylated (Figure 5a–c). Further experiments using short hairpin RNA (shRNA) directed against AMPK were performed. AMPK expression was down-regulated in primary neurons using shRNA directed against the α1 and α2 AMPK catalytic subunits (shAMPK) (Figure 5d,e). In these conditions, shAMPK reduced the phosphorylation of ACC following SA, confirming its inhibitory effect on AMPK signaling (Figure 5d). Moreover, following SA, shAMPK led to a significant reduction of PKA-substrate and CREB phosphorylation as compared to the control non-targeting shRNA (shNT) (Figure 5d,f,g), thus validating the results obtained with Cc.

Altogether, these results show for the first time that AMPK activation cross-talks with the PKA pathway to regulate the expression of IEGs following SA. 

## 3. Discussion

Changes in the expression of IEGs is an important process mediated by synaptic activity that is necessary for the conversion of short-term memory to long-term memory. With the present study, we extended on our previous data to determine the mechanism by which AMPK activity following synaptic activation led to the expression of IEGs. Here, we showed that SA led to the activation of the PKA/CREB pathway in an AMPK-dependent manner.

Whether AMPK directly or indirectly regulated the PKA/CREB pathway remains to be explored. However, in a previous report, we demonstrated that AMPK was required during SA to maintain intracellular ATP levels [20], therefore, it is possible that AMPK indirectly regulated the PKA pathway via controlling ATP levels. ATP, indeed, is converted by AC into cAMP, the second messenger that regulates PKA. Therefore, it is possible that the drop of ATP levels, due to AMPK inhibition, could lead to a parallel decrease of cAMP production, and eventually to a decrease of the signaling systems dependent on PKA.

Interestingly, our data showed that membrane-bound AC are not responsible for PKA activation following SA. Rather, it is the unconventional sAC (ADCY10) that is involved. sAC is distributed throughout the cytoplasm and in cellular organelles including the nucleus and mitochondria. Its functions are distinct from those of the transmembrane AC. For instance, it is insensible to G-proteins and forskolin regulation. However, in neuronal cells sAC activity can also be activated by intracellular Ca^2+^ elevations that increase its affinity for ATP [23] but also by bicarbonate anions (HCO_3_^−^) that increase the enzyme’s V_max_. Importantly, HCO_3_^−^ can be metabolically generated within the cells under the action of carbonic anhydrases (CA), hence sAC activity can be modulated by metabolically generated HCO_3_^−^ within the mitochondria [25,26,27]. Thus, mitochondrial metabolism regulated by AMPK could be another level of regulation of sAC, and hence cAMP production. Finally, we cannot exclude the possibility that AMPK could also regulate in a more direct fashion the sAC, through phosphorylation for instance. Finally, recent results have reported that activation of the mitochondrial cannabinoid receptor (mtCB1) caused inhibition of mitochondrial sAC, which resulted in reduction of PKA-dependent regulation of mitochondrial respiration, and eventually amnesic effects [28]. 

It is also interesting to note that AMPK was reported to be regulated by phosphorylation on Ser^485/491^ on its catalytic subunits, respectively to α1 and α2. This phosphorylation occurs in response to agents that elevate intracellular cAMP, such as forskolin and isobutylmethylxanthine, and is likely to be mediated by PKA. These agents, however, act via membrane bound AC. Therefore, further investigations would be required to determine whether the sAC could also regulate PKA-mediated phosphorylation of AMPK. Interestingly, this phosphorylation of AMPK could be implicated in attenuating its activity given that it is associated with a down-regulation of its phosphorylation on Thr^172^ [29]. Further, in adipocytes, PKA was found to phosphorylate Ser^173^ on the AMPK α subunit to regulate lipolysis in response to PKA-activating signals [30]. Altogether these studies show that PKA can negatively regulate AMPK activity. It is therefore possible that PKA activation could in return repress AMPK activity, which could be an interesting mechanism to recover a basal AMPK activity state following SA.

Importantly, our data ([20], this study) suggest that neuronal energetic status may influence the formation of long-term memory. The hypothesis that AMPK influences these processes by maintaining ATP levels raises the question of long-term memory formation in an energetic stress environment. Metabolic disorders such as obesity and diabetes are characterized by peripheral metabolic dysfunction, but also cognitive deficits [31], elevated neurodegerenative disease risk, especially for Alzheimer’s disease [32], and have recently been associated with central metabolic perturbations [33]. Interestingly, other studies have shown that several neurodegenerative diseases, including Alzheimer’s disease, are not only associated with hypometabolism, but also to an activation of AMPK [34].

In conclusion, our study adds a player in the induction of signaling pathways involved in the regulation of the expression of IEGs, and hence memory formation. Altogether, our data suggest that through energy levels regulation, AMPK might indirectly control the activity of other signaling pathways, including those regulated by the second messenger cAMP.

## 4. Materials and Methods 

### 4.1. Chemicals and Reagents/Antibodies

Antibodies directed against AMPKα (1/1000, Rabbit), ACC (1/1000, Rabbit), phospho-Ser^79^ACC (1/1000, Rabbit), phospho-PKA substrate (RRXS*/T*) (1/2000, Rabbit), phospho-AMPK substrate (1/1000, Rabbit), and phospho-Ser^133^CREB (1/1000, Rabbit) were obtained from Cell Signaling technology (Danvers, MA, USA). Anti phospho-Thr^172^AMPKα (1/1000, Rabbit), CREB (1/500, Rabbit), Arc (1/500, Mouse), c-Fos (1/500, Mouse), and EgrI (1/500, Rabbit) antibodies were from Santa-Cruz (Dallas, TX, USA). Anti-actin (1/15 000, Mouse) antibody was from BD Bioscience (Franklin Lakes, NJ, USA). HRP-coupled secondary antibodies directed against the primary antibodies’ hosts were obtained from Cell Signaling technology. Bicuculline (Bic), H 89, PKI 14-22 amide, NKY 80, SQ 22536, and KH 7 were purchased from Tocris (Bristol, UK), 4-aminopyridine (4-AP) was purchased from Sigma (St Louis, MO, USA), and Compound C (Cc) was from Santa Cruz (Dallas, TX, USA).

### 4.2. Primary Neuronal Cell Culture and Treatments

All animal experiments were performed according to procedures approved by the local Animal Ethical Committee following European standards for the care and use of laboratory animals (agreement APAFIS#4689-2016032315498524 v5 from CEEA75, Lille, France; approved on Oct 11, 2016). Primary neurons were prepared as previously described [35]. Briefly, fetuses at stage E18.5 were obtained from pregnant C57BL/6J wild-type female mice (The Jackson Laboratory, Bar Harbor, ME, USA). Forebrains were dissected in ice-cold dissection medium composed of Hanks’ balanced salt solution (HBSS) (Invitrogen, Carlsbad, CA, USA) supplemented with 0.5 % *w*/*v* D-glucose (Sigma, St Louis, MO, USA) and 25 mM Hepes (Invitrogen, Carlsbad, CA, USA). Neurons were dissociated and isolated in ice-cold dissection medium containing 0.01 % *w*/*v* papain (Sigma, St Louis, MO, USA), 0.1 % *w*/*v* dispase (Sigma, St Louis, MO, USA), and 0.01 % *w*/*v* DNaseI (Roche, Rotkreuz, Switzerland), and by incubation at 37 °C for 15 min. Cells were spun down at 220 x g for 5 min at 4 °C, resuspended in Neurobasal medium supplemented with 2% B27, 1 mM NaPyr, 100 units/mL penicillin, 100 μg/ml streptomycin, and 2 mM Glutamax (Invitrogen, Carlsbad, CA, USA). For Western blots experiments, 12-well plates were seeded with 500,000 neurons per well and for RT-qPCR experiments, 6-well plates were seeded with 1,000,000 neurons per well. Fresh medium was added every 3 days (1:3 of starting volume). Cells were then treated and collected between DIV 14 to 17. For shRNA transduction, shRNA vectors from the TRC-Mm1.0 (Mouse) library, shAMPK α1 (CloneID:TRCN0000024000) shAMPK α2 (CloneID:TRCN0000024046), and non-targeting control shRNA (RHS6848) were obtained from Dharmacon, Lafayette, CO, USA. For the lentiviral production, HEK 293T cells were transfected for 72 h before collecting the supernatant as previously described [20]. Supernatant was concentrated using Amicon^®^ Ultra 15-mL Centrifugal Filters (EMD Millipore, Burlington, MA, USA). Primary neuronal cultures were transduced with both AMPK α1 and AMPK α2 shRNA or the non-targeting shRNA at DIV 7, 7 days before performing experimentation.

### 4.3. Immunoblotting

For Western blot (WB) analysis, 15 µg of proteins from total cell lysates were separated in 8–16% Tris-Glycine gradient gels and transferred to nitrocellulose membranes. Membranes were then blocked in 5% fat-free milk in Tris Buffer Saline-0.01% Tween-20, and incubated with specific primary antibodies overnight at 4 °C. Proteins were thereafter detected via the use of Horseradish Peroxidase-conjugated secondary antibodies and electrochemiluminescence detection system (ThermoFisher Scientific, Waltham, MA, USA). The Western blot bands corresponding to proteins of interest, or smears for phosphorylated-PKA substrate, were analyzed using the FIJI software v1.51n [36].

### 4.4. Quantitative Real-Time RT-PCR for the Expression of IEGs

Total RNA was isolated using the NucleoSpin^®^ RNA kit (Macherey-Nagel, Düren, Germany) according to the manufacturer’s instructions. One microgram of total RNA was reverse-transcribed using the Applied Biosystems High-Capacity cDNA reverse transcription kit (ThermoFisher Scientific, Watham, MA, USA). Real-time quantitative reverse transcription polymerase chain reaction (qRT-PCR) analyses were performed using Power SYBR Green PCR Master Mix (ThermoFisher Scientific, Watham, MA, USA) on a StepOneTM Real-Time PCR System (ThermoFisher Scientific, Watham, MA, USA) using the following primers: *β-actin* forward: 5′-CTAAGGCCAACCGTGAAAAG-3′, reverse: 5′-ACCAGAGGCATACAGGGACA-3′; *Arc* forward: 5′-GGTGAGCTGAAGCCACAAAT-3′, reverse: 5′-TTCACTGGTATGAATCACTGCTG-3′; *EgrI* forward: 5′-AAGACACCCCCCCATGAA-C-3′, reverse: 5′-CTCATCCGAGCGAGAAAAGC-3′; and *c-Fos* forward: 5′-CGAAGGGAACGGAATAAG-3′, reverse: 5′-CTCTGGGAAGCCAAGGTC-3′. The thermal cycler conditions were as follows: hold for 10 min at 95 °C, followed by 45 cycles of a two-step PCR consisting of a 95 °C step for 15 s followed by a 60 °C step for 25 s. Amplifications were carried out in triplicate, and the relative expression of target genes was determined by the ΔΔCT method using β-actin for normalization.

### 4.5. Statistical Analyses

All statistical analyses were performed using GraphPad Prism (Prism 5.0d, GraphPad Software Inc, La Jolla, CA, USA).

## Abbreviations

AC	Adenylyl cyclase
ACC	Acetyl-CoA carboxylase
AMPK	AMP-activated protein kinase
Arc	Activity-regulated cytoskeleton-associated protein
Bic/4-AP	Bicuculline/4-aminopyridine
cAMP	3′,5′-cyclic AMP
Cc	Compound C
CREB	cAMP response element binding
DIV	Days in vitro
GPCRs	G protein-coupled receptors
IEGs	Immediate Early Genes
KO	Knock-out
PKA	cAMP-dependent protein kinase
SA	Synaptic activation
sAC	Soluble adenylyl cyclase
shRNA	Short hairpin RNA
